# Flame Retardancy and Dispersion of Functionalized Carbon Nanotubes in Thiol-Ene Nanocomposites

**DOI:** 10.3390/polym13193308

**Published:** 2021-09-28

**Authors:** Jiangbo Wang

**Affiliations:** School of Materials and Chemical Engineering, Ningbo University of Technology, Ningbo 315211, China; jiangbowang@nbut.edu.cn; Tel.: +86-0574-87081240

**Keywords:** thiol-ene, carbon nanotubes, polysilicone, functionalization, flame retardancy, dispersion

## Abstract

A polysilicone flame retardant (PA) was synthesized and covalently grafted onto the surface of carbon nanotubes (CNTs) via amide linkages to obtain modified CNTs (CNTs-PA). The grafting reaction was characterized by Fourier transform infrared (FTIR) spectroscopy, X-ray photoelectron spectrometer (XPS), Transmission electron microscopy (TEM) and Thermogravimetric analysis (TGA), and the resultant CNTs-PA was soluble and stable in polar solvents Chloroform. Thiol-ene (TE)/CNTs-PA nanocomposites were prepared via Ultraviolet curing. The flame retardancy of thiol-ene nanocomposites was improved, especially for the heat release rate. Moreover, the results from Scanning electron microscopy (SEM) and Dynamic mechanical thermal analysis (DMTA) showed that the CNTs-PA improved the dispersion of CNTs in thiol-ene and enhanced the interfacial interaction between CNTs-PA and thiol-ene matrix.

## 1. Introduction

Thiol-ene (TE) polymerization systems have been widely used in recent years for their rapid polymerization, low volume shrinkage, overall uniformity and insensitivity to oxygen inhibition as compared to traditional photopolymerized networks [1,2,3,4]. However, it demands to develop flame retardant systems to reduce the fire hazards in some applications since the Ultraviolet (UV) cured thiol-ene products are generally flammable. Flame retardant polymers can be prepared by the addition of flame retardant additives to the polymer or by attaching flame retardant compounds to the polymer with chemical bonds [5,6].

Carbon nanotubes (CNTs) have attracted great interest among researchers in both academia and industry, due to their unique structural, mechanical, electronic, and thermal properties [7,8,9,10]. Particularly, CNTs have been used as a candidate of flame retardant additive in polymer matrix composites. Kashiwagi et al. [11,12] reported the systematic study on the flammability of polymer/CNTs composites, and significant reduction in heat release rate (HRR) was observed after CNTs were incorporated into poly (methyl methacrylate) and polypropylene at a very low loading. However, the insolubility and the aggregation of CNTs in polymer matrix have severely limited their applications [13,14,15,16]. To solve these problems, various organic, inorganic, and organometallic structures have been used for functionalizing CNTs. Among them, the long chains of flame-retardant polymer were gaining particular interest [17,18,19,20,21,22].

Moreover, the addition of conventional flame retardants at present is at more than 10 wt%, and for some flame retardants the concentration is as high as 40 wt%. Higher concentrations of flame retardants not only adversely affect the material properties (especially the mechanical properties), but also, they are not conducive to the recycling of polymers. For example, halogen flame retardants and phosphorus flame retardants are toxic and harmful having many adverse effects on the environment. The carbon nanotube flame retardant has very good flame retardant effect at a low concentration (2 wt%). Further, what is more, it is environment-friendly, non-polluting, and at the end of life can be recycled many times.

In this work, carbon nanotubes (CNTs) were grafted with polysilicone (PA), and the FTIR, XPS, TEM and TGA were used to characterize the structure of modified CNTs. It is anticipated that the covalent graft of PA onto CNTs can improve the compatibility and dispersion of CNTs in TE matrix, and accordingly promote the flame retardancy of TE/CNTs nanocomposites.

## 2. Materials and Methods

### 2.1. Materials

Concentrated sulphuric acid (H_2_SO_4_, 98%), nitric acid (HNO_3_, 65–70%), pyridine (99.5%), phenyltriethoxysilane (PTES), tetramethylammonium hydroxide (TMAOH), N,N-dimethylformamide (DMF, 99%) and tetrahydrofuran (THF) were all purchased from Alfa Aesar Chemical Reagent Co., Ltd. (Tewksbury, MA, USA). Chloroform (CHCl_3_) and thionyl chloride (SOCl_2_) were supplied by Fisher Scientific Chemical Co. (Waltham, MA, USA). CNTs (outer diameter 10–20 nm, inner diameter 5–10 nm, length 10–30 μm) synthesized by chemical vapor deposition were purchased from Chengdu Organic Chemistry Co. Ltd., Chinese Academy of Science (Chengdu, Sichuan, China). Ethyl alcohol (EtOH), (3-aminopropyl)triethoxysilane (APTES), pentaerythritol allyl ether (TAE) and 2,2-dimethoxy-2-phenylacetophenone (DMPA) were obtained from Sigma-Aldrich Co. Ltd. (St. Louis, MO, USA). Trimethylolpropane tris(3-mercaptopropionate) (3T) was purchased from Bruno Bock Chemische Fabrik Gmblt & Co. (Marschacht, Germany) and used as received.

### 2.2. Synthesis of Polysilicone (PA)

**Scheme 1:**
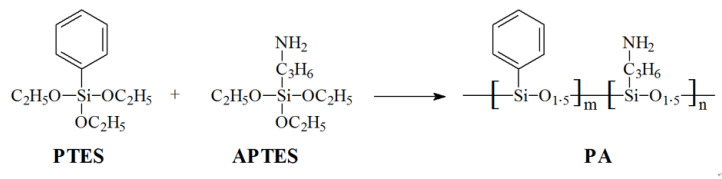
Synthesis route of PA.

Distilled water (25 mL), EtOH (75 mL) and TMAOH (1 mL) were mixed in a 250 mL flask under stirring (Scheme 1). The mixture of PTES and APTES at 95:5 molar ratios was added into the above solution, maintaining 10% weight percentage. The stirring was stopped after 8 h, and the solution was aged at room temperature overnight. Precipitated condensate was collected by decantation of most clear supernatant, washed by vacuum filtration with distilled H_2_O/EtOH (1/3 by volume), and then washed again in pure EtOH. The rinsed polysilicone powder (PA) was dried thoroughly under vacuum for 20 h at room temperature [23].

### 2.3. Functionalization of CNTs

**Scheme 2:**
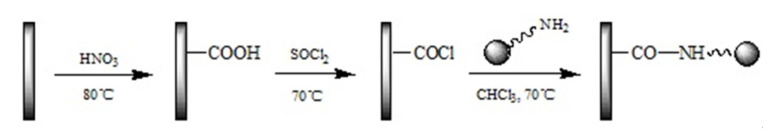
Illustration for the functionalization of the CNTs with PA.

The CNTs-COCl was synthesized as follows (Scheme 2): The mixture of CNTs, HNO_3_ (30 mL) and H_2_SO_4_ (90 mL) was sonicated at 50 °C for 2 h. After termination of reaction, it was allowed to cool down to room temperature. The mixture was diluted with a large amount of deionized water, followed by a vacuum-filtering through a nylon film (0.22 μm, Sangon Biotech (Shanghai) Co., Ltd., Shanghai, China). The obtained solid CNTs-COOH, in which polar carboxyl groups were introduced into the convex surface of CNTs, was washed with deionize water until the aqueous layer reached neutral, and then was vacuum-dried at 80 °C for 12 h. The reaction mixture of CNTs-COOH (200 mg), SOCl_2_ (20 mL) and DMF (1 mL) was sonicated at 50 °C for 1 h, and then refluxed at 70 °C for 24 h. After that, the temperature was risen to 120 °C and CNTs-COCl was obtained after residual SOCl_2_ was removed by the reduced pressure distillation.

The CNTs-PA was synthesized as follows: PA (400 mg) and pyridine (1 mL, as cat.) were added into the suspension of CNTs-COCl (100 mg) and DMF (50 mL) under the protection of nitrogen, and the mixture was reacted at 70 °C for 24 h. After cooling to room temperature, the dark solution was filtered and washed to remove unreacted PA. The target product CNTs-PA after vacuum-dryness at 80 °C for 24 h was obtained.

### 2.4. Preparation of Thiol-Ene Nanocomposites

**Scheme 3:**
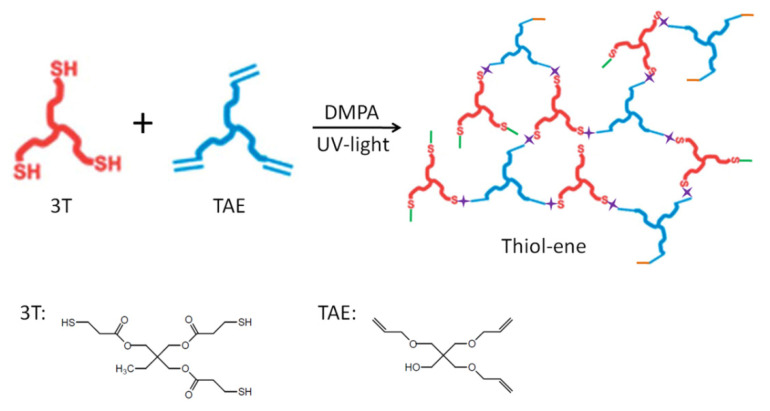
Thiol-ene polymerization reaction.

Briefly, the TE/CNTs-PA nanocomposites were prepared as follows (Scheme 3): Photoinitiator (DMPA, 1 wt%) was dissolved in thiol (3T) and mixture sonicated for 30 min. Then, TAE (1:1, equivalent ratio to thiol), CNTs-PA (2 wt%) were added into mixture and new slurry was mixed well using glass rod (~ 1 min). After further mixing and removal of bubbles using a sonicator (30 min), homogenous mixtures were drawn down onto glass substrates using drawndown bar. Films were cured using 10 passes under a Fusion UV Curing Line system using a D bulb (400 W/cm^2^ with belt speed of 3 m/min and 3.1 W/cm^2^ irradiance). For comparison, pure TE and 2 wt% CNTs/TE (TE/CNTs) nanocomposites were also prepared at same processing condition.

### 2.5. Characterization and Measurement

FTIR spectra of the dried samples were recorded using a Broker Equinox-55 IR spectrometer (Digilab Inc., Hopkinton, MA, USA) at a resolution of 2 cm^−1^ with 20 scans. The samples were mixed with potassium bromide and pressed to a disc, which was used to measure. X-ray photoelectron spectroscopy (XPS) was carried out in a Thermo Scientific ESCALAB 250Xi X-ray photoelectron spectrometer (Thermo Fisher Scientific Inc., Waltham, MA, USA) equipped with a mono-chromatic Al Kα X-ray source (1486.6 eV). The surface morphology of carbon nanotubes was observed by JEOL JEM-2100F (TEM, JEOL Ltd., Akishima-shi, Tokyo, Japan) transmission electron microscopy (TEM). Cone calorimeter measurement was performed on an FTT cone calorimeter (Fire Testing Technology Ltd., East Grinstead, West Sussex, UK) according to ASTM E1354 with heat flux of 50 kW/m^2^. The dimensions of each specimen were 100 × 100 × 3 mm^3^. Thermogravimetric analysis (TGA) measurement was carried on a TA instrument Q5000 (TA Instrument Corp., New Castle, DE, USA) thermogravimetric analyzer. The sample (about 10 mg) was heated from 50 °C to 700 °C at a 10 °C/min heating ramp rate in nitrogen atmosphere. The samples were coated with a conductive gold layer and examined by scanning electron microscopy (SEM) using FEI Quanta 200 environmental (FEI company, Hillsboro, OR, USA) scanning electron microscope. Dynamic mechanical thermal analysis (DMTA) was determined using a Rheometric Scientific SR-5000 (Rheometric Scientific Inc., West Yorkshire, UK) dynamic mechanical analyzer and the data were collected from −40 °C to 20 °C at a scanning rate of 5 °C/min. The tensile strength and elongation at break were measured at room temperature by a universal testing machine (DXLL-20000) (Shanghai Dejie Instrument Inc., Shanghai, China) with a crosshead speed of 5 mm/min. For each sample, five specimens were measured.

## 3. Resultsand Discussion

### 3.1. Structural Characterization

**Figure 1:**
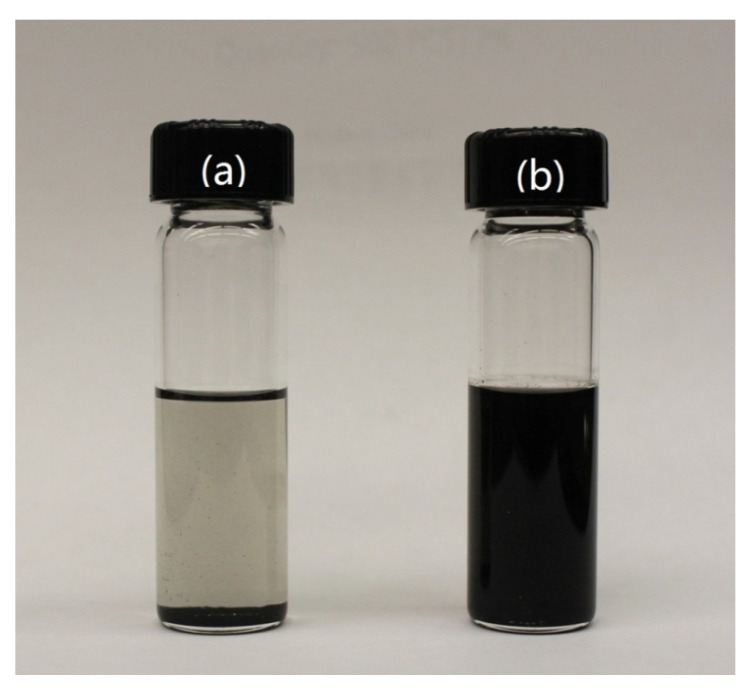
Digital photos of the solubility of CNTs (**a**) and CNTs-PA (**b**) in CHCl_3_.

In this study, CNTs-PA was synthesized by the chemical reaction between acyl chloride group from CNTs and amino group from PA. The successful synthesis of the target product CNTs-PA was confirmed by the solubility, FT-IR, XPS, TEM and TGA measurements. Dispersion of pristine CNTs into organic solvent was very difficult, even after the sample has been subjected to ultrasonication. However, the functionalized CNTs usually show a much higher solubility or better dispersion in solvents. As shown in Figure 1, it is clear that CNTs is insoluble in CHCl_3_, and there was much sedimentation of nanotubes at the bottom of a small bottle. For comparison, the CNTs-PA is soluble in CHCl_3_ and forming a stable black solution even after 1 month. This indicates that CNTs-PA possess a higher degree of miscibility than CNTs due to the presence of PA functional groups on the surface, as already proved above.

**Figure 2:**
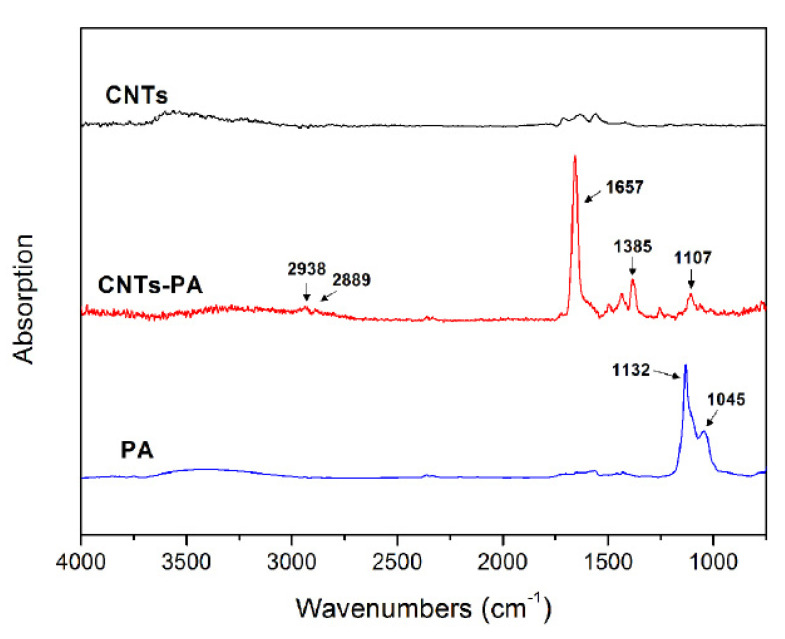
FTIR spectra of CNTs, PA and CNTs-PA.

As shown in Scheme 2, PA is grafted on the surface of CNTs.FTIR spectra can be used to reveal the reaction between CNTs and PA. Figure 2 shows the FTIR spectra of pristine CNTs, PA and CNTs-PA. Comparing with CNTs and PA, the strong peak at 1657 cm^−1^ in CNTs-PA curve was observed and corresponded to the stretching vibration of the C=O group. The signal of symmetrical Si–O–Si bonds in the PA shown in the CNTs-PA was characterized by the stretching bands at 1107 cm^−1^. Moreover, the successful functionalization of PA on the surface of CNTs provide new asymmetric stretching and symmetric stretching of –CH_2_– at 2938 and 2889 cm^−1^ to the CNTs-PA spectra, where there is also a weak C–N stretching at 1385 cm^−1^. These FTIR results verify the existence of PA molecules grafted to the CNTs through the amide bonds.

**Figure 3:**
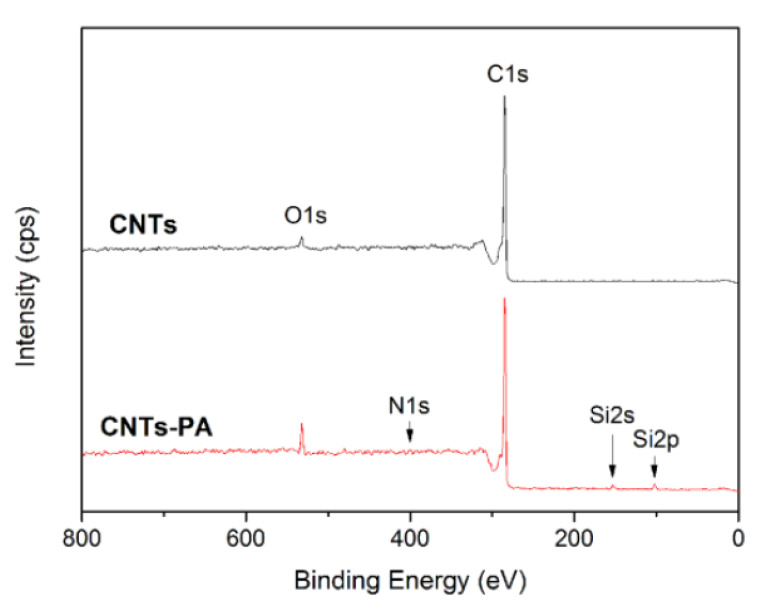
XPS survey spectra of CNTs and CNTs-PA.

XPS analysis was utilized to determine the chemical composition of the CNTs functionalized by PA. Figure 3 showed XPS survey spectra of CNTs and CNTs-PA, and the binding energies of the elements are summarized in Table 1. It can be obtained that CNTs exhibited C1s and O1s peaks at 285 and 532 eV, respectively, and the pristine CNTs is composed of carbon atoms (96.25%) and some oxygen atoms (3.75%). For comparison, there are C, O, Si, and N atoms on the surface of CNTs-PA and the intensity of the O1s peak of CNTs-PA is higher than that of CNTs. The atomic percentage of the silicon is 1.08% and the nitrogen is 0.59% in CNTs-PA. The above XPS survey spectra further supported the results from solubility measurement and infrared spectra.

**Figure 4:**
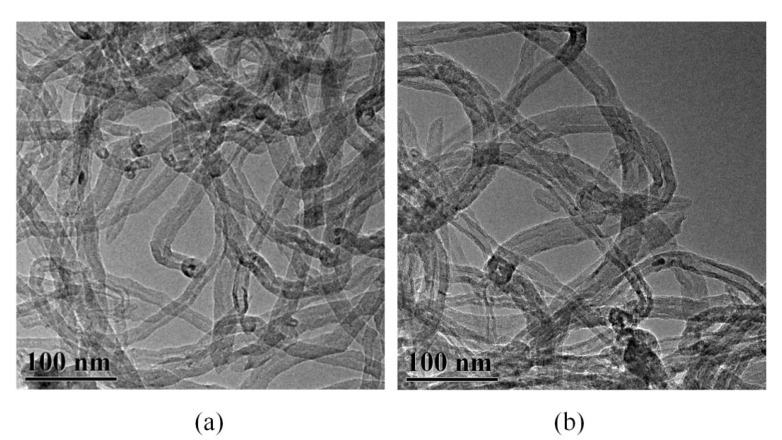
TEM images of CNTs (**a**) and CNTs-PA (**b**).

In order to obtain the morphology of evidence, the comparison for the microstructures of CNTs and CNTs-PA was presented in Figure 4. Figure 4a displays a typical TEM image of CNTs, showing very smooth and clear surface without any extra phase adhering to them. However, the CNTs-PA shown in Figure 4b appears stained with an extra phase that is presumed to mainly come from the grafted PA molecules. Furthermore, the increase of tube diameters is discerned from CNTs-PMDA in Figure 4b. This indicates that PA has been successfully grafted to the surface of CNTs.

**Figure 5:**
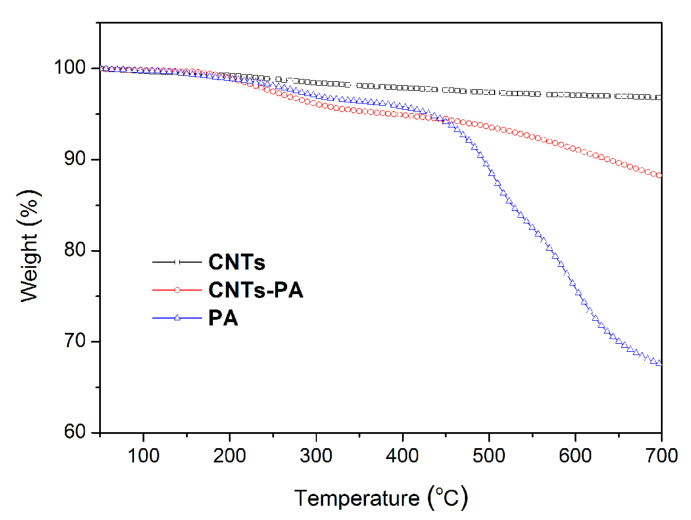
TGA curves of CNTs, PA and CNTs-PA.

TGA measurement provides further evidence for the polysilicone functionalization of CNTs.The thermal stability of pristine CNTs, PA and CNTs-PA was studied by TGA under N_2_ atmosphere, as shown in Figure 5. It can be obtained that the pristine CNTs hardly decomposes and about 96.78 wt% residues are left at 700 °C. In contrast, the CNTs-PA is less thermally stable, and the amount of the CNTs residues is about 88.17 wt% at 700 °C. Moreover, the relative amount of grafted PA onto CNTs can be estimated by the TGA analysis. The differences in the weight loss between CNTs, PA and CNTs-PA at 700 °C exhibit that the content of covalently grafted PA is 29.4 wt%.This result is consistent with the carbon tube micrographs obtained from TEM measurement, indicating that the morphology of carbon tube has changed after grafting reaction.

### 3.2. Flame Retardancy

**Figure 6:**
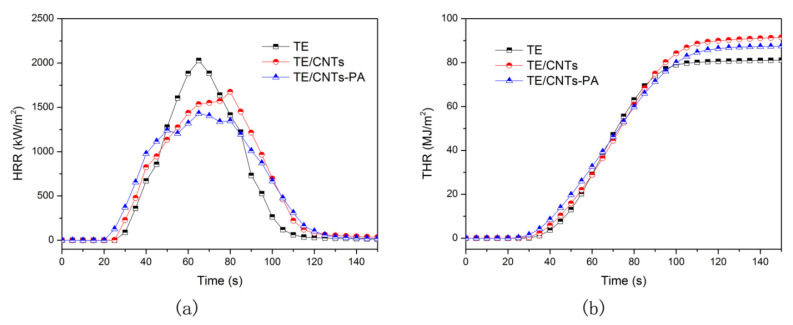
HRR (**a**) and THR (**b**) curves of TE and TE/CNTs-PA nanocomposites.

The experimental results of cone calorimeter at a flux of 50 kW/m^2^ are presented in Figure 6. The heat release rate (HRR) is a very important parameter and can be used to express the intensity of a fire. A highly flame-retardant system normally shows a low HRR value. It can be found that the peak of heat release rate (PHRR) for the pure TE reaches a value of 2030.1 kW/m^2^, and which presents very sharp HRR curve. As is clearly evident, the incorporation of pristine CNTs considerably reduced the PHRR of TE (around 17.5% reduction). Further reduction of PHRR from 2030.1 kW/m^2^ to 1432.1 kW/m^2^ is observed for CNTs-PA with TE, which is reduced by 29.5%. Meanwhile, compared with the TE/CNTs, the total heat release (THR) value of TE/CNTs-PA is decreased by 4.4%, as shown in Figure 6. The decrease of HRR and THR indicates that the incorporation of CNTs-PA into composites can restrict the fire development.

### 3.3. Dispersionand Mechanical Properties

**Figure 7:**
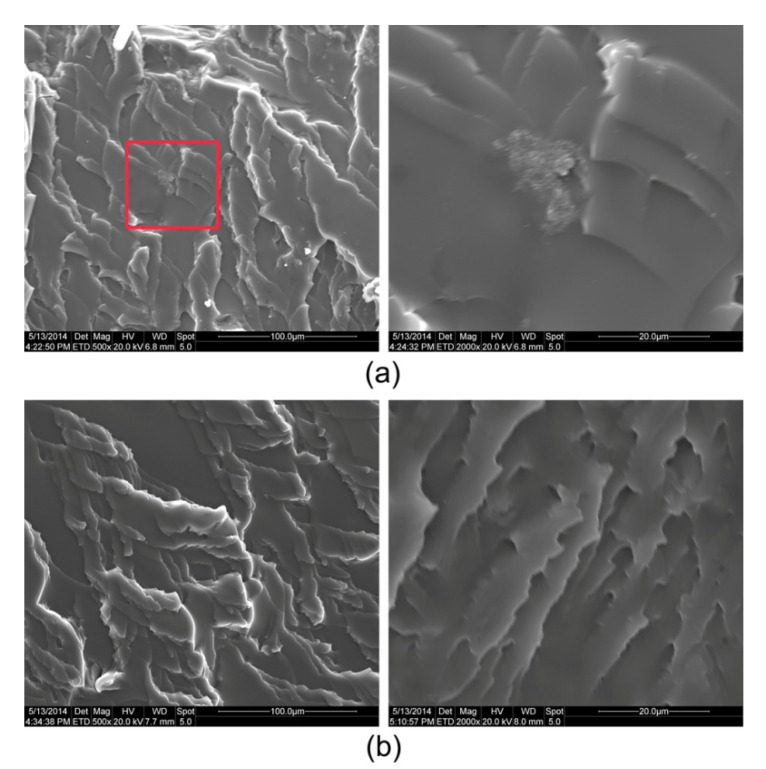
SEM images of TE (**a**) and TE/CNTs-PA (**b**) nanocomposites.

For evaluation of the dispersion of CNTs in polymer matrix, the SEM images of the fracture surface of TE nanocomposites were shown in Figure 7. It could be seen that the pristine CNTs had some aggregation in the TE matrix (Figure 7a), which was due to the poor compatibility between CNTs and TE matrix. However, after grafted with PA, CNTs-PA dispersed in the TE matrix homogeneously and no obvious aggregation was observed in Figure 7b. Thus, the surface modification was an effective method to prevent the agglomeration of CNTs in polymer matrix.

**Figure 8:**
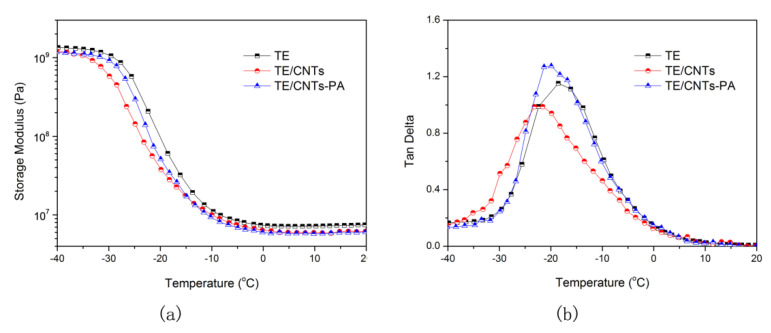
Dynamic storage modulus (**a**) and tan δ (**b**) of TE and TE/CNTs-PA nanocomposites.

The dynamic storage modulus (E’) and the loss tangent (tan δ) versus the temperature of TE nanocomposites was presented in Figure 8. It can be seen that the incorporation of CNTs decreases the E’ of the nanocomposites slightly (Figure 8a). However, after functionalization of CNTs with PA, the E’ of the TE/CNTs-PA nanocomposites increase dramatically to 51.3 MPa at −20 °C, which is 35.7% larger than the E’ of TE/CNTs. This rapid rise occurs because CNTs-PA are more dispersed and had stronger interfacial adhesion with the TE matrix than the CNTs. Moreover, as shown in Figure 8b, the glass transition temperature (T_g_) of the TE/CNTs and TE/CNTs-PA nanocomposites decreased slightly compared with that of pure TE. It is well known that in polymer matrix composites, the T_g_ of the polymer matrix depends on the free volume of the polymer, which is related to the affinity between the filler and the polymer matrix. The incorporation of CNTs may interfere with the interaction of the TE polymer chains in composites, leading to a large free volume beyond that of the TE matrix, and also resulting in the lower T_g_ of TE/CNTs composites [20,24].

Therefore, the results from SEM images and DMTA revealed that the PA grafting significantly improves the dispersion of CNTs in the TE matrix.

**Figure 9:**
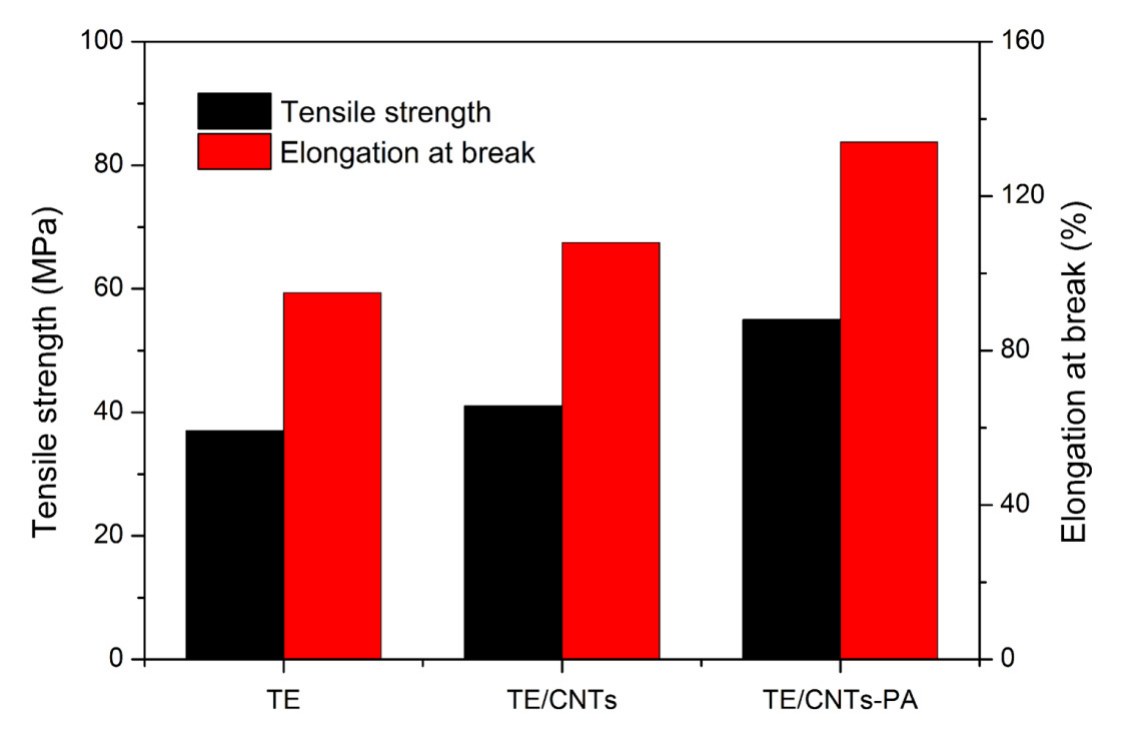
Mechanical properties of TE nanocomposites.

Figure 9 shows the mechanical properties of TE nanocomposites. It can be seen that the tensile strength and elongation at break of pure TE were 37 MPa and 95%, respectively. After adding carbon nanotubes, the tensile strength and elongation at break of TE were increased to 41 MPa and 108%, respectively, which were higher than those of pure TE. Furthermore, the mechanical properties of TE nanocomposite were further improved by the addition of CNTs-PA. Compared with pure TE, the tensile strength and elongation at break of TE/CNTs-PA were increased by 48.6% and 41.1%, respectively. The results showed that the improvement of mechanical properties of TE nanocomposite by carbon nanotubes is closely related to the compatibility and dispersion of carbon tubes in polymer matrix, and CNTs-PA can effectively improve the mechanical properties of TE nanocomposite.

## 4. Conclusions

In this work, a facile and efficient method was used to functionalize carbon nanotubes (CNTs) with a polysilicone flame retardant (PA) via amide linkages. The results from FTIR, XPS, TEM and TGA showed that PA has been covalently grafted onto the surface of CNTs, and the resultant CNTs-PA was soluble and stable in polar solvents CHCl_3_. With the incorporation of CNTs-PA in thiol-ene (TE), a significant improvement in flame retardancy of TE nanocomposites was achieved and the reduction of PHRR from 2030.1 kW/m^2^ to 1432.1 kW/m^2^ was observed for CNTs-PA with TE. Moreover, the results from SEM and DMTA revealed that the functionalization of CNTs with PA improved the dispersion of CNTs in TE and the interfacial interaction between CNTs-PA and TE matrix was simultaneously enhanced. Furthermore, compared with pure TE, the tensile strength and elongation at break of TE/CNTs-PA were increased by 48.6% and 41.1%, respectively.

## Figures and Tables

**Table 1:** Element composition of CNTs and CNTs-PA.

Element (At.%)	CNTs	CNTs-PA
C	96.25	93.65
O	3.75	4.68
Si	-	1.08
N	-	0.59

## Data Availability

The data used to support the findings of this study are available from the corresponding author upon request.
